# 3D Polymer-Based 1 × 4 MMI Splitter

**DOI:** 10.3390/nano12101749

**Published:** 2022-05-20

**Authors:** Tomas Mizera, Peter Gaso, Dusan Pudis, Martin Ziman, Anton Kuzma, Matej Goraus

**Affiliations:** 1 Department of Physics, Faculty of Electrical Engineering and Information Technology, University of Zilina, Univerzitna 1, 01026 Zilina, Slovakia; gaso@fyzika.uniza.sk (P.G.); goraus@fyzika.uniza.sk (M.G.); 2 University Science Park of the University of Zilina, Univerzitna 1, 01026 Zilina, Slovakia; 3 Faculty of Electrical Engineering and Information Technology, Institute of Electronics and Photonics, Slovak University of Technology in Bratislava, Ilkovicova 3, 81219 Bratislava, Slovakia; martin.ziman@stuba.sk (M.Z.); anton.kuzma@stuba.sk (A.K.)

**Keywords:** multimode interference splitter, 3D splitter, polymer, direct laser writing

## Abstract

Due to the increasing trend of photonic device miniaturisation, there is also an increased need for optical splitting in a small volume. We propose a smart solution to split light in three dimensions (3D). A 3D optical splitter based on multimode interference (MMI) for the wavelength of 1550 nm is here designed, simulated, fabricated and optimised for splitting at 1550 nm. We focus also on the possibility of its direct integration on an optical fibre. The design is focused on the use of 3D laser lithography based on the direct laser writing (DLW) process. The output characteristics are investigated by near-field measurement, where we confirm the successful 1 × 4 splitting on a 158 µm long MMI splitter.

## 1. Introduction

The ever-increasing demand for miniaturisation has led to an increasing number of photonic devices for on-chip integration. The resulting network of optical connections on a chip in an integrated photonic circuit needs, as building blocks, efficient components for beam splitting and shaping [1,2].

Splitting devices are essential components and most often use two approaches to split the signal. The first types are Y-branch splitters or their cascade configuration. This type of splitter has seen an increase in popularity in recent years, mainly in planar configurations [3]. Moreover, a 3D polymer-based Y-branch splitter was recently described by Gaso et al. [4]. Another type uses the interference phenomenon for optical splitting and is known as a multimode interference (MMI) splitter. MMI-based splitters have advantages such as low losses, wide manufacturing tolerances, low polarisation dependence and mainly small-volume splitting, while the Y-branch splitters need a high-radius geometry [5,6].

Recently, the development of MMI splitters has moved to the forefront because of their attractiveness in photonic integrated circuits (PICs). The basic MMI splitter is an asymmetric MMI 1 × 2 silicon-on-insulator (SOI)-based power splitter [7]. The authors demonstrated the strengths of the MMI splitter, making it potentially suitable for monitoring performance on a sizeable silicon-PIC chip. Furthermore, a polarisation beam splitter with a high extinction ratio based on multimode interference (MMI) has been designed and experimentally demonstrated on an SOI platform [8]. As advantages, the authors identified that the device has a large manufacturing tolerance with an MMI width of ±40 nm and length of ±1500 nm. Similarly, the proposed structure can be used in silicon photonic integrated circuits for polarisation division multiplexing. Another MMI splitter for PIC is based on a 3 × 1 MMI combiner and a slot waveguide structure, which can use slot waveguide technology to reduce losses [9].

Splitters made with photonic crystals are also worth mentioning. An example is the design of a dielectric inverse photonic crystal structure that couples line-defect waveguide propagating modes into highly directional beams of controllable directionality, presented by Tasolamprou et al. [10]. The structure utilises a triangular lattice made of air holes drilled in an infinitely thick Si slab, and it is designed for operation in the near-infrared and optical regime. The structure generates well-defined, spatially and spectrally isolated beams, and may serve as a frequency splitting component designed for operation in the near-infrared regime and, in particular, in the telecom optical wavelength band. Another example of the use of a photonic crystal, this time in square geometry, can be found in an article by Makwan et al. [11]. In addition to their use in PICs, they are widely used in optical networks and play a crucial role in optical communication systems. MMI splitters were successfully applied as filters [12], temperature sensors [13], splitters [14,15] and couplers [16]. These MMI splitters have been made on an inorganic crystal, semiconductor and polymeric material basis. Their arrangement uses planar geometry, which significantly limits the design and method of splitting. Polymers have received much attention as a building material for photonic elements in recent years. This is due to the low price, the relatively simple technological process and the excellent stability of the current polymers [17].

Modern three-dimensional (3D) technologies based on polymers have provided unconventional solutions for photonic components [18]. In general, polymer-based photonic devices use polymers with absorption losses of a few dB/cm, limiting output parameters such as the Q-factors and insertion losses of polymer-based photonic devices, and cannot be compared to standard silicon photonic components. However, they offer unlimited designs and fast and inexpensive one-step technologies.

To our knowledge, no concept of an MMI-based 3D beam splitter coupled to a single-mode fibre (SMF) has been published. A possible solution offers polymer photonics based on the 3D laser writing process. Weigel et al. used the MMI principle and proposed the connection of four waveguide layers with three cascading 1 × 1 3D MMIs [19]. The basic building block consists of a 1 × 1 3D MMI with dimensions of (10.4 × 1.4 × 173) μm^3^, which was described by M. Nuck et al. [20]. The interference part and the connecting waveguides are based on a polymer core and a polymer shell with an index contrast Δ*n* = 0.03 at 1550 nm. The authors documented the structure losses and their dependence on the wavelength. They concluded that the multilayer connection showed a minimum loss on the chip of 2.5 dB at a wavelength of 1517 nm. This multilayer structure is essential for the new complex routing without waveguide crossing and for the optical switching matrices. Nuck et al. [21], in turn, proposed a switching matrix consisting of two cascaded 3D 4 × 4 MMIs to connect four single-mode input waveguides to each single-mode output waveguide in a 3D arrangement. The authors demonstrated an insertion drop below 9.3 dB, including a fibre-chip connection loss and a 6 dB internal loss. The mentioned that MMI splitters and photonic devices with novel 3D designs demonstrate the many advantages of polymeric materials for use in photonic integrated polymer-based devices and also have shown the lack of a polymer-based 3D MMI splitter in different splitting configurations.

This paper presents a novel 1 × 4 optical signal splitter arranged in 3D geometry working at a wavelength of 1550 nm and based on a polymer. We designed a polymer structure capable of using the MMI effect to split the optical signal at 1550 nm. With regard to the laser writing technology and fibre coupling, we processed a 3D 1 × 4 MMI splitter and we measured its optical properties and the characteristics of the output field. The proposed 3D geometry is original and new and, at the same time, considerably reduces the length of the splitter with respect to the other published planar optical splitters.

### Theory of MMI Splitters

The main design task of MMI splitting is to set the appropriate dimensions of the interference part. This interference part is a multimode waveguide and works on the principle of the self-imaging effect, which is an essential feature of multimode interference. The superposition of the propagating modes of the multimode waveguide generates a field distribution along the MMI splitter. The characteristic parameter in the case of MMI splitters is their beat length—*L_MMI_*—the length at which the output interference maximum is a mirror image of the input. This interference pattern is then repeated periodically with the periodicity of the beat length. The beat length is given by Equation (1) [22,23,24]:

(1) $$L_{MMI}=\frac{\pi }{\beta_{0}-\beta_{1}}\approx \frac{4n_{c}W^{2}}{3\lambda_{0}}$$

where $\beta_{0}$ and $\beta_{1}$ are the propagation constants of the fundamental and the first-order lateral modes, $n_{c}$ is the effective refractive index of the MMI core, W is the effective width, and $\lambda_{0}$ is the wavelength of the input signal. For defined width W at given wavelength $\lambda_{0}$, we obtain the splitting of the input electromagnetic wave into the desired number of interference maxima, N.

The proposed concept of the 3D MMI splitter is based on the IP-Dip polymer as a core material. IP-Dip is a negative photoresist typical for the laser writing process, which, in this context, creates the coupling part, the interference part and even the supporting structure. As a cladding, we consider the surrounding air.

## 2. 3D MMI Splitter Design for Configuration 1 × 4

The 3D polymer MMI beam splitter 1 × 4 was designed and simulated for an operating wavelength at 1550 nm. The simulations were performed using the RSoft Tool [25]. For the broader use of single-mode optical fibres (SMF), the splitter has been designed to be compatible with SMF. The splitting part cross-section was chosen on a square basis to suppress polarisation and wavelength-dependent losses.

**Figure 1:**
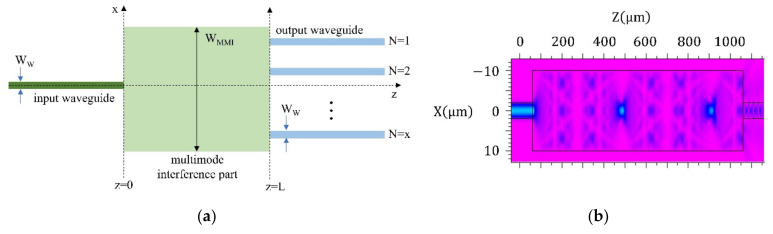
(**a**) Basic arrangement of MMI splitter and (**b**) simulation of electromagnetic wave distribution in the 3D MMI splitter for a (20 × 20) μm^2^ square base taken at the central part of the splitter.

Firstly, we analysed the MMI splitting along the interference part to find the important interference centres and the beat length. Figure 1a shows the most general arrangement of MMI splitters, with the basic parameters of such splitters. General MMI splitters consist of (i) an input waveguide (dark green) of a waveguide width $W_{W}$, (ii) a multimode waveguide as the interference part (light green) of the length L and of the width $W_{MMI}$ and (iii) the corresponding output waveguides (blue). The length  L of the MMI splitting part determines the number of outputs.

The simulation in Figure 1b shows the electromagnetic wave intensity distributions in the proposed 3D MMI splitter. The structure was designed with a square base with (20 × 20) µm^2^. The interference part itself is designed from the polymeric material IP-Dip, whose refractive index is $n_{c}$ = 1.53 surrounded by air $n_{cl}=1$ [26]. The simulation also demonstrates the interference pattern along the proposed structure. In the proposed design with a base of (20 × 20) μm^2^, the characteristic beat length is approximately 490 μm.

**Figure 2:**
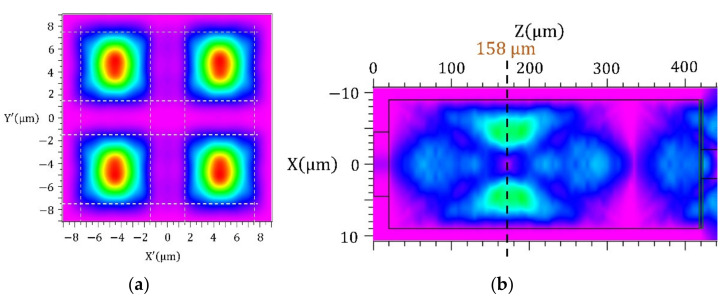
Simulation of electromagnetic wave intensity distribution (**a**) at the end of the MMI part (**b**) along part of the MMI. The white lines indicate the width determination of the interference centres.

The next step of the design was to determine the exact length *L* of the interference part to achieve 4 interference maxima and so the 1 × 4 MMI configuration. From the simulations (Figure 2b), we found an optimal design of a square base with a width of $W_{MMI}=18\;\mu m$, and a length of the interference part L=158 μm. For the length *L*, we analysed the optical field distribution at the end of the proposed MMI structure (Figure 2a). This confirmed the existence of four symmetrical interference maxima with estimated dimensions of (6 × 6) µm^2^, which fully met our requirements for further signal outcoupling.

Furthermore, careful optimisation showed the dependence between the shape of the input mode and the shape of the output field. Since the requirement was to connect the splitter to the SMF, it was necessary to ensure a suitable shape of the input field. Due to this, an input waveguide (taper) was added to the structure to ensure the uniformity of the input signal. Wang et al. have addressed similar tapers for SMF and multimode fibres (MMF). In their article, they considered fabrication techniques and the stability analysis of SMF-/MMF-based differently tapered optical fibre structures [27]. In this work, fabrication techniques and optimisation of SMF and multimode fibre-based differently tapered optical fibre structures are discussed.

**Figure 3:**
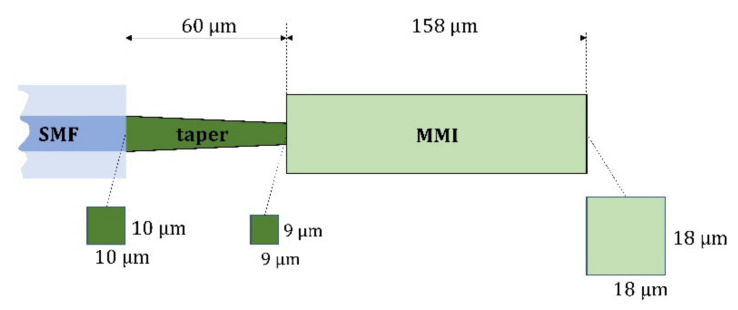
The MMI splitter geometry of the optical splitting part with corresponding dimensions.

The dimensions of this coupling part are (10 × 10) µm^2^ on the SMF side and (9 × 9) µm^2^ for the MMI part, and the taper length is 60 µm. These dimensions were chosen with respect to the DLW technology limit, which is 300 μm in height, and this limit should include the complete length of the designed MMI splitter. The resulting geometry of the whole splitter with corresponding dimensions is shown in Figure 3.

**Figure 4:**
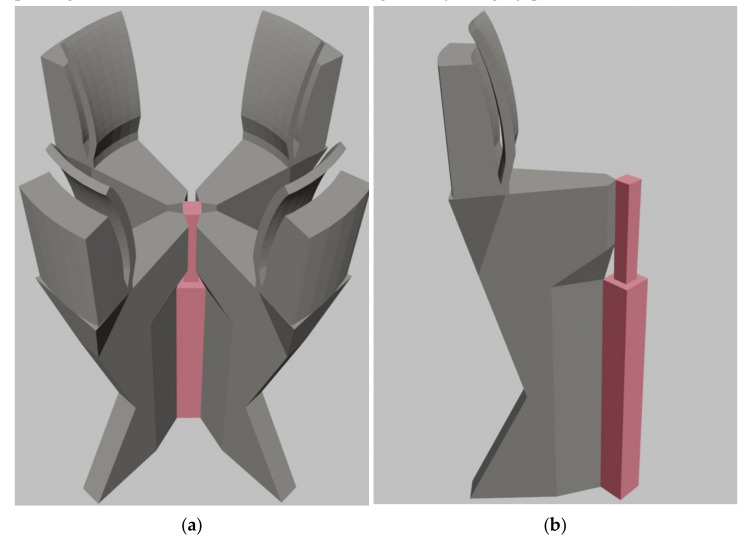
Three-dimensional MMI splitter design: (**a**) whole construction with mechanical clamps for SMF fixation (grey parts); (**b**) 3D view of partly revealed supporting mechanical structure (grey) together with the optical splitter part (pink).

This final geometry was used for the design of the optical splitting part of the MMI splitter. It consists of the MMI multimode interference waveguide (light green), the input waveguide taper (dark green) and the input SMF. However, many of processed experiments showed that the 3D beam structure in such an arrangement is too fragile. Therefore, we focused on producing a more robust MMI splitter. We have developed a stable supporting mechanical structure, as shown in Figure 4 by the grey parts.

Figure 4 demonstrates a detailed view of the connection of the MMI splitting part (pink) with the supporting mechanical structure (grey) through thin polymer junctions. The input port for the SMF consists of four clamps with sufficient flexibility to ensure the guidance and attachment of the SMF.

## 3. Fabrication of 3D Splitter

**Figure 5:**
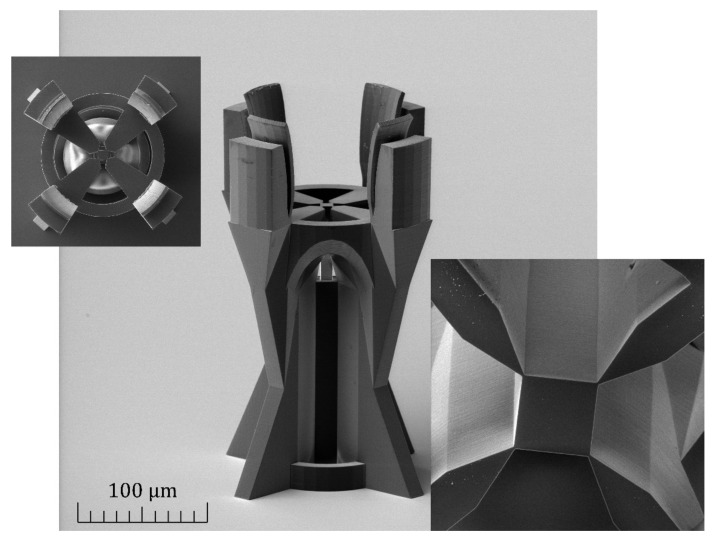
SEM image of the 3D 1 × 4 MMI splitter, prepared from IP-Dip polymer, with inserted images of top view from output side (top left) and detail of output MMI part coupled to supporting construction.

For the fabrication of the MMI splitter, the IP-Dip photoresist (Nanoscribe GmbH & Co. KG, Eggenstein-Leopoldshafen, Germany) was used in a single-step process based on direct laser writing (DLW) using two-photon polymerisation (TPP). The commercial Nanoscribe Photonic Professional GT laser lithography system (Nanoscribe GmbH & Co. KG, Eggenstein-Leopoldshafen, Germany) based on DLW was the crucial technology for the fabrication of the MMI splitter. The laser focused on the IP-Dip photoresist and formed a focal spot with a lateral resolution reaching 200 nm and a vertical resolution of 600 nm. The system used an Er-doped femtosecond frequency-doubled fibre laser emitting pulses at a wavelength of 780 nm, with an approximately 100 MHz repetition frequency and a pulse duration of 150 fs, and a maximum power of 50 mW [28]. A high-resolution galvanometer mirror system scanned the laser beam in the sample plane. The IP-Dip photoresist was used in immersion laser lithography (DILL) mode, where the lens was immersed in a liquid photoresist. We used a scanning speed of 10,000 μm/s and a laser power of 20 mW. In the exposed volume, the IP-Dip photoresist polymerised due to the TPP. After the polymerisation process, the sample was developed in propylene glycol monomethyl ether acetate (PGMEA). The whole structure was prepared on a glass substrate (Figure 5). The scanning electron microscope (SEM) image showed a very smooth surface of high optical quality.

Great precision was required for the mechanical coupling of the SMF to the input part of the fabricated MMI splitter, and therefore, we used a micromechanical stage for approaching the SMF fibre. During this approaching process, the MMI structure was placed upside down, as is shown in Figure 5, and SMF was introduced from above. The whole approaching process was monitored by an objective and camera. The glass substrate was mechanically removed after this procedure.

## 4. Results

**Figure 6:**
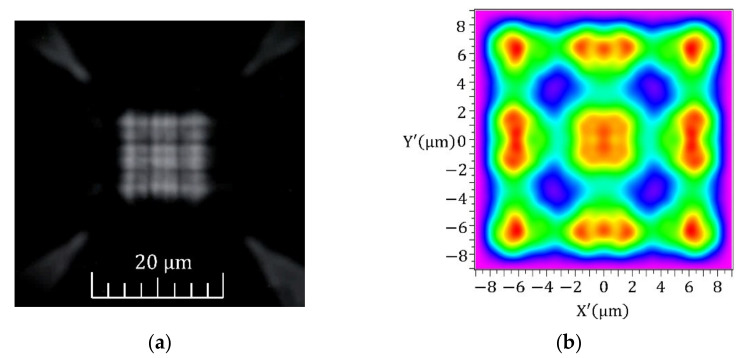
(**a**) Optical microscope image of interference pattern generated by 940 nm excitation and (**b**) simulated optical field distribution for the wavelength of 940 nm.

Optical properties were analysed with an optical microscope and by scanning the modal structure at the splitter output using a highly resolved near-field scanning optical microscope (NSOM). The overall optical guiding and splitting were first examined by near-infrared light propagation. As an excitation light source, we used a light-emitting diode (LED) coupled to the optical fibre, with a central emission wavelength at *λ* = 940 nm. The optical microscope image of the splitter is shown in Figure 6a. The output detail shows a square-based output part fixed via thin junctions connected to mechanical arms. As can be seen, only a small part of the propagating light leaked from the guiding part, which demonstrates low-loss guiding. At the same time, we can observe the interference pattern at the output of the splitter corresponding to the interference for the *λ* = 940 nm. Moreover, the simulation shows a similar interference pattern at the output, which demonstrates the correct functionality of the interference part for different wavelengths too.

In further research, we focused on the characterisation of the modal distribution across the output part using highly resolved NSOM. NSOM uses a very sharp optical fibre probe with a lateral resolution better than 100 nm, collecting the optical field at the output splitter’s surface in very close proximity [29]. Light from a fibre-coupled laser source with a central wavelength of 1550 nm was directly connected to the input waveguide. The near-field distribution near the output was measured in transmission mode. The detection optical fibre probe was connected to a femtowatt InGaAs detector (1100–1700 nm) and amplified by a LockIn amplifier (Stanford Research Systems, Sunnyvale, CA, USA) [30].

**Figure 7:**
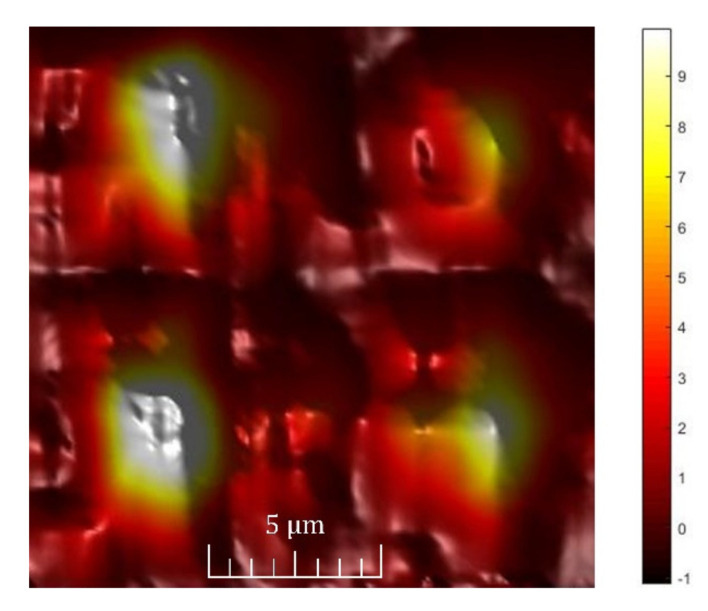
Near-field distribution of waveguide output for the MMI splitter excited by a laser at 1550 nm.

The near-field optical field distribution at the output part of the MMI splitter is shown in Figure 7. From the modal distribution, we can see the existence of four dominant interference maxima. The same distribution was also simulated and is documented in Figure 2.

**Figure 8:**
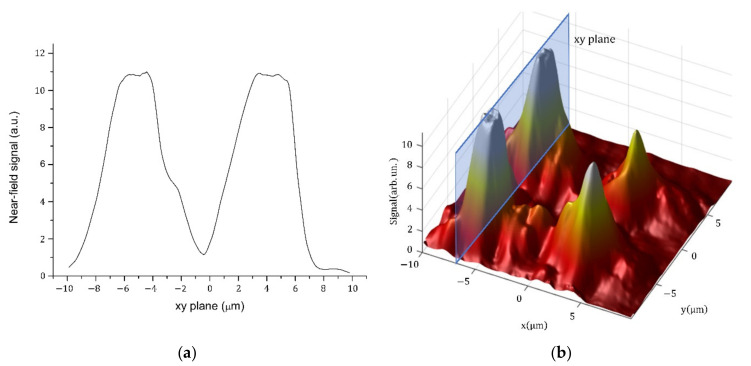
The cross-section of the optical field intensity at the output part: (**a**) along the two planes, as shown in (**b**) 3D interpretation of the intensity distribution.

The detailed analysis of the output optical field distribution was analysed from the cross-sections of the output signal (Figure 8). Figure 8b shows a 3D interpretation of the optical field intensity distribution. The profiles of the individual cross-sections’ maxima correspond well to the simulated results. We determined the field averages of the individual modes from the cross-sections. All four maxima had a mode field diameter (MFD) of approximately (6 × 6) µm^2^, while the simulation assumed that MFD was (6 × 6) µm^2^. Moreover, the simulation correlated well with the measured shape of the optical field at the output. From the 3D analysis, we can conclude that the individual modes measured at the output of the MMI splitter do not have the same intensity as the output part and are strongly dependent on the input coupling. Weak coupling asymmetry at the input causes the misbalancing of the intensity between individual modes. However, the presented optical splitter prepared purely from IP-Dip polymer offers an interesting means to split radiation in a very short distance using the 3D configuration.

In the final analysis, we focused on the insertion loss determination. Firstly, we roughly estimated an insertion loss from BPM simulation. The signal at single output was attenuated by −6.77 dB, which means −0.75 dB attenuation of all four outputs. The simulation neglected the surface roughness of the waveguides, the fabrication imperfections on the interfaces and the losses due to the thin-wall bonding to the mechanical structure of the 3D MMI splitter. However, the simulated insertion losses were compared with the measurement of the signal outputs over all four outputs. We measured an overall loss of −2.59 dB. According to the near-field distribution from the image in Figure 7, we attributed the individual loss in individual channels as follows: −7.44 dB for both the strongest outputs and −10 dB and −10.45 dB for both low signal outputs, respectively. The signal distribution between the outputs depends on the input SMF coupling and needs more expertise and input guiding optimisation.

## 5. Conclusions

The achieved results document the first successful realisation of a 3D MMI splitter based on a polymer. The optical field distribution showed nearly ideal splitting, with four evident maxima. Moreover, the output field profiles corresponded well with the simulated results. We achieved a mode field diameter up to the field averages of the individual modes from the cross-sections. All four maxima achieved MFD of approximately (6 × 6) µm^2^, which corresponded well with the simulation, and similarly, the measured shape reflected well the simulated optical field at the output. Weak asymmetry of the output modes was closed by asymmetric input coupling. This sensitivity of the input coupling was caused by the short input waveguide part and could not be further improved in this configuration. Generally, the presented 3D 1 × 4 MMI splitter offers a novel and smart realisation of light splitting in small volumes.

## Data Availability

Not applicable.
